# Reconstruction of the middle hepatic vein using a vein graft from the resected portion of the liver

**DOI:** 10.1186/s40792-020-01057-8

**Published:** 2020-11-01

**Authors:** ShiWei Yang, DongDong Han, Liang Wang, Lei Gong, CanHong Xiang

**Affiliations:** Department of Hepatobiliary Surgery, Beijing Tsinghua Changgung Hospital, Tiantongyuan, Changping, Beijing, China

**Keywords:** Reconstruction of middle hepatic vein, Autologous vein, Intrahepatic cholangiocarcinoma

## Abstract

**Background:**

The middle hepatic veins are often infiltrated by intrahepatic cholangiocarcinoma. Reconstruction of the hepatic vein plays a critical role in preserving more of the residual liver volume and reducing the risk of postoperative liver failure in extreme hepatectomy. We here report a novel way to reconstruct middle hepatic vein by using vessel grafts from wasted liver.

**Case presentation:**

Case 1: A 64-year-old man was diagnosed with intrahepatic cholangiocarcinoma. The bifurcation and left branch of the portal vein were stenosed, and the root of the middle hepatic vein was infiltrated by the tumor. An extended left hepatectomy was performed, the portal vein was resected and reconstructed, and the middle hepatic vein was reconstructed by anastomosing the proximal left hepatic vein to the distal middle hepatic vein. Case 2: A 69-year-old woman was diagnosed with intrahepatic cholangiocarcinoma. The tumor was located in the left lobe of the liver and the left and middle hepatic veins were infiltrated by the tumor. An extended left hepatectomy was performed, and the left portal vein was used as a vein graft to reconstruct the middle hepatic vein. Both of the two patients’ postoperative ultrasound showed vessel graft patency.

**Conclusion:**

Using a vein graft from the resected portion of the liver to reconstruct the middle hepatic vein was a useful technique and showed good result.

## Background

Normal segmental liver function depends on whether there is sufficient blood flow. Insufficient blood flow into or out of the liver can lead to liver dysfunction. The middle hepatic veins are often infiltrated by intrahepatic cholangiocarcinoma due to its biological characteristics. Reconstruction of the hepatic vein plays a critical role in preserving more of the residual liver volume and reducing the risk of postoperative liver failure in extreme hepatectomy. Vessel grafts for reconstruction of the middle hepatic vein include an autologous saphenous vein [1, 2], the internal jugular vein [3], the ovarian vein [4–6], the umbilical vein [6–8], an artificial vessel [9], and an allogeneic vessel [10, 11]. The use of vessel grafts from wasted liver has advantages of less damage and more similar character for middle hepatic vein reconstruction. This report describes the methods used to reconstruct the middle hepatic vein using a vein graft from the resected portion of the liver, which is rarely reported before.

## Patient 1

**Fig. 1:**
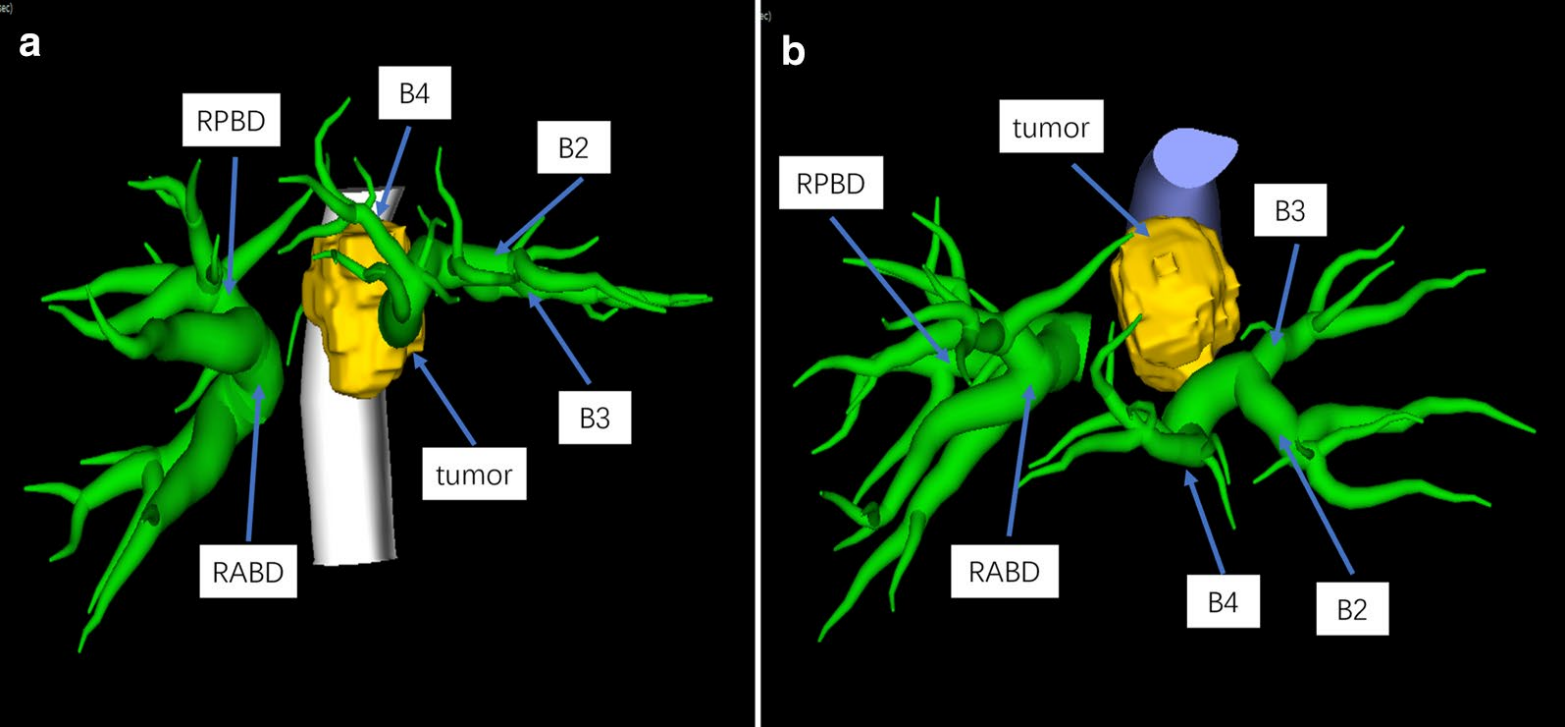
Three-dimensional reconstruction of the tumor and bile duct shows the tumor was located at the hilar bile duct, and the left and right intrahepatic bile ducts were dilated. **a** Shows the relationships between bile duct with tumor in sagittal section. **b** Shows the relationships between bile duct with tumor in transverse section. *B2* bile duct of segment 2, *B3* bile duct of segment 3, *B4* bile duct of segment 4, *RABD* bile duct of right anterior liver lobe, *RPBD* bile duct of right posterior liver lobe

**Fig. 2:**
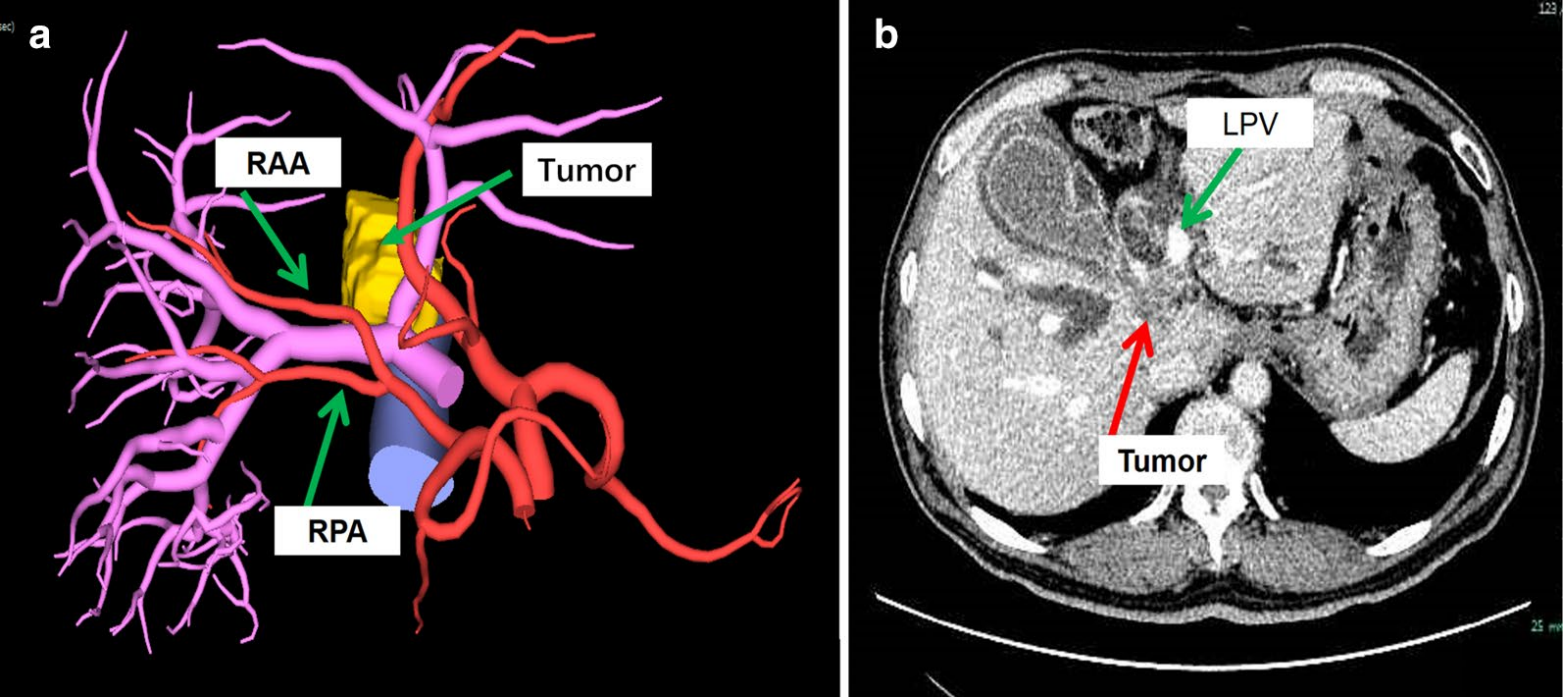
Location of tumor and vessels: **a** the three-dimensional reconstruction of the tumor and artery, the right branch was closely related to the tumor, and the right hepatic artery was from the superior mesenteric artery and was not affected. **b** The CT scan which shows the tumor was located at the bifurcation of the portal vein, the left branch of the portal vein was compressed. *RAA* right anterior hepatic artery, *RPA* right posterior hepatic artery, *LPV* left portal vein

**Fig. 3:**
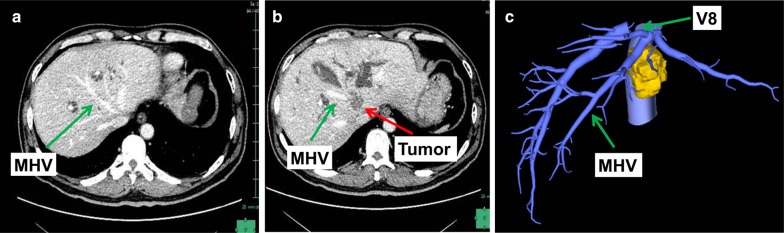
Relationship between tumor and hepatic veins: **a** and **b** are CT scan which shows that the tumor infiltrated the root of the middle hepatic vein, and the left hepatic vein was not infiltrated. **c** The three-dimensional reconstruction of the tumor and hepatic vein. *MHV* middle hepatic vein, *V8* hepatic vein of segment 8

**Fig. 4:**
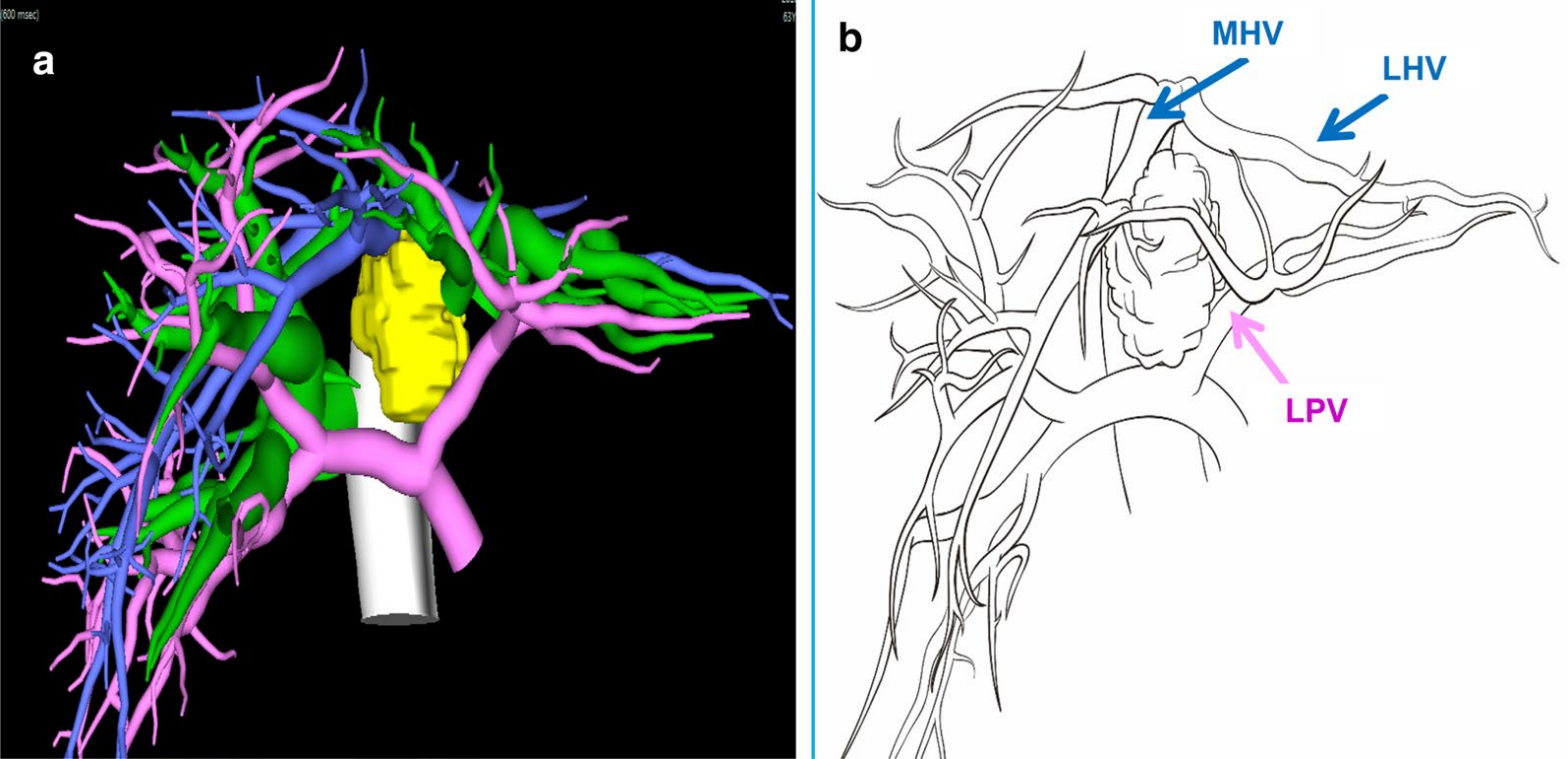
Fusion images of the tumor and bile ducts: **a** the three-dimensional reconstruction of vessels and tumor. **b** Shows the relationship between tumor with portal vein and hepatic vein. The tumor infiltrated the bifurcation of portal vein and left branch, bifurcation of hilar bile duct, and the middle hepatic vein. *MHV* middle hepatic vein, *LHV* left hepatic vein, *LPV* left portal vein

**Fig. 5:**
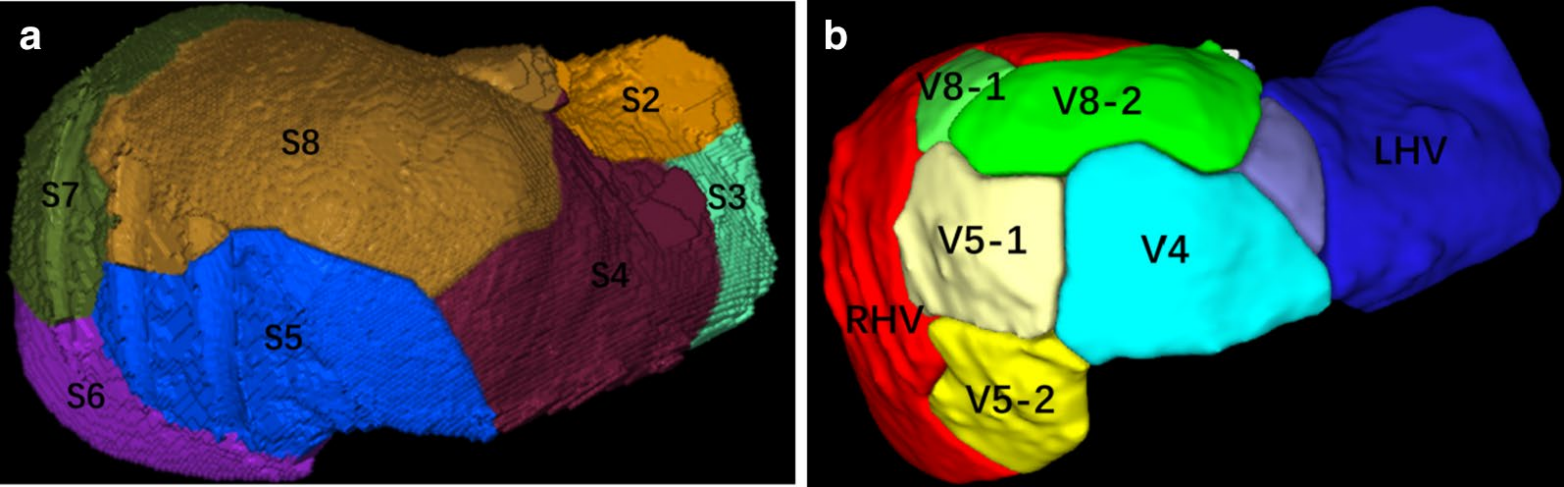
Three-dimensional reconstruction of the liver segments: **a** shows the area dominated by portal vein branch: S2: 99 mL (5.9%), S3: 74 mL (4.4%), S4: 237 mL (14.2%), S5: 186 mL (11.2%), S6: 182 mL (10.9%), S7: 409 mL (24.5%), S8: 479 mL (28.8%). **b** Shows the area dominated by hepatic vein branch: RHV: 630 mL (37.8%), LHV: 243 mL (14.6%), V8-1: 108 mL (6.5%), V8-2: 233 mL (14.0%), V5-1: 122 mL (7.3%), V5-2: 140 mL (8.4%), V4: 150 mL (9.0%). S2: segment 2, S3: segment 2, S4: segment 4, S5: segment 5, S6: segment 6, S7: segment 7, S8: segment 8, LHV: left hepatic vein, RHV: right hepatic vein, V4: hepatic vein of segment 4, V5-1: a branch of MHV which drained part one of segment 5, V5-2: a branch of MHV which drained part two of segment 5, V8-1: a branch of MHV which drained part one of segment 8, V8-2: a branch of MHV which drained part two of segment 8. *MHV* middle hepatic vein

**Fig. 6:**
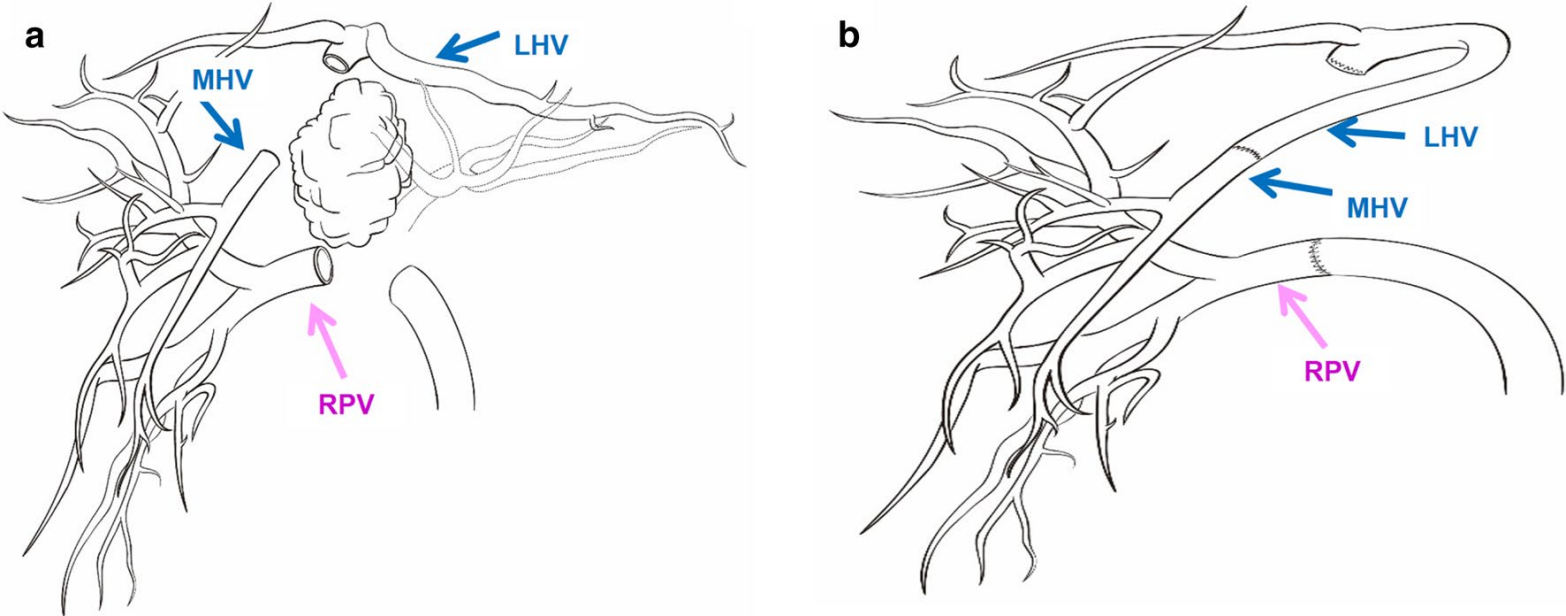
Schematic diagram of the operation: **a** shows the location to remove MHV and RPV. **b** Shows the way to reconstruct MHV and RPV. The left hepatic vein was used for reconstruction of the middle hepatic vein, because it was far away from the tumor which ensured the R0 resection of the tumor and the entirety of hepatic blood inflow and outflow. *MHV* middle hepatic vein, *LHV* left hepatic vein, *RPV* right portal vein

**Fig. 7:**
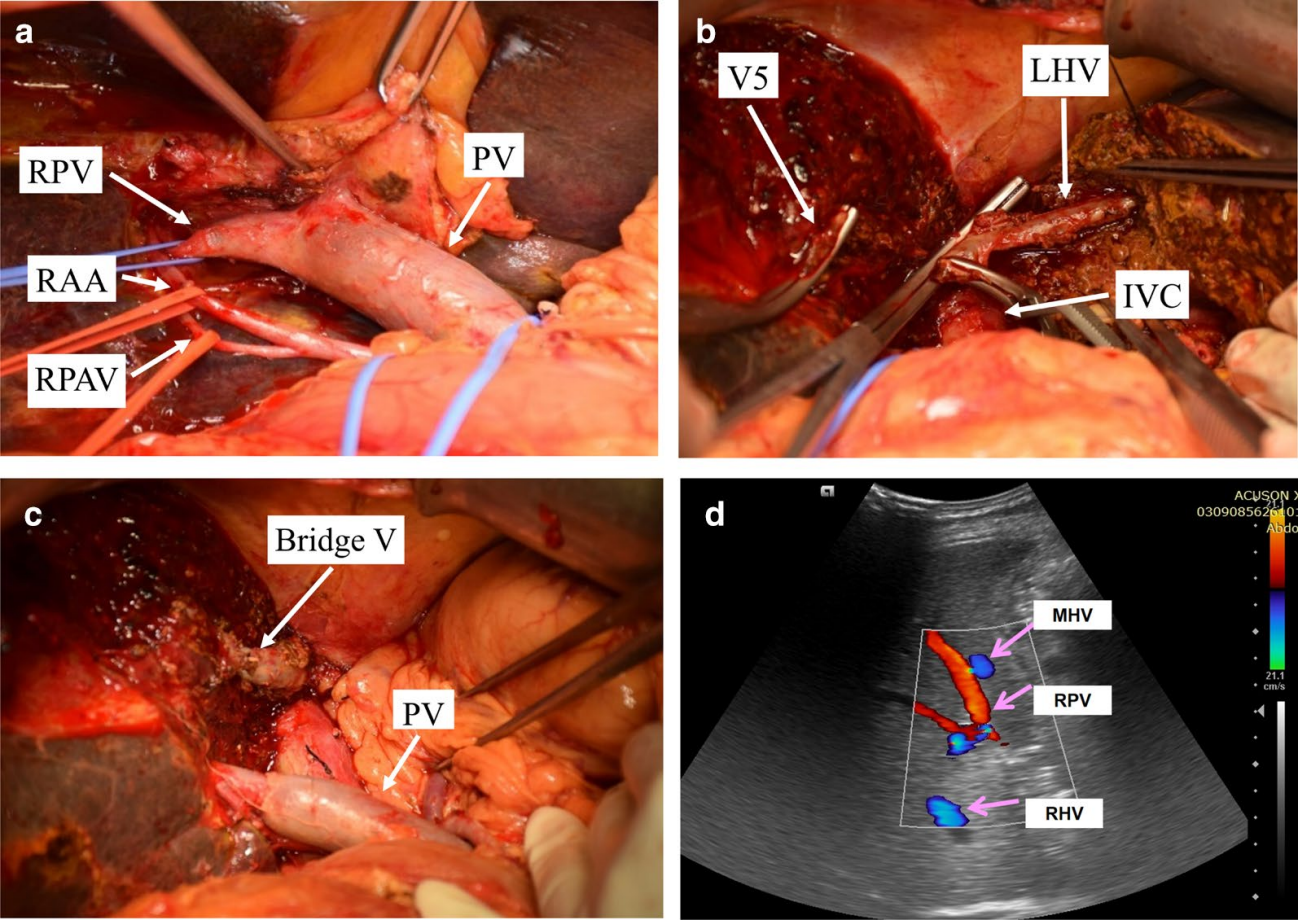
Intraoperative hepatic vascular management: **a** skeletonization of right hepatic artery, portal vein. The bifurcation of portal vein was infiltrated by the tumor. **b** The root of the middle hepatic vein was infiltrated, after dissecting part of left hepatic vein from its root and rotated its direction, the main trunk of the middle hepatic vein was reconstructed with end to end anastomosis. **c** Reconstruction images of portal vein and hepatic vein. **d** Ultrasound on postoperative day 60 shows good blood flow in the right branch of the portal vein and middle hepatic vein. *RPV* right portal vein, *PV* portal vein, *MHV* middle hepatic vein, *LHV* left hepatic vein, *RAA* right anterior artery, *RPA* right posterior artery, *IVC* inferior vena cava, *Bridge V* bridge vein

The patient was a 64-year-old man who was admitted for jaundice 10 days prior. Blood tests indicated a total bilirubin level of 297.68 umol/L, a direct bilirubin level of 247.68 umol/L, an alanine aminotransferase level of 360.3 U/L, an aspartate aminotransferase level of 273.6 U/L, a total protein level of 49.9 g/L, an albumin level of 28.5 g/L, a CEA level of 115.8 ng/mL, and a CA 19–9 level of 246.2 U/mL. Abdominal CT revealed a lesion in the hilar bile duct with indistinct margins; the tumor was moderately enhanced during the arterial phase, and the intrahepatic bile ducts were dilated (Fig. 1). The bifurcation and left branch of the portal vein were stenosed, and the root of the middle hepatic vein was affected by the lesion (Figs. 2, 3, 4). The patient was diagnosed with intrahepatic cholangiocarcinoma. Total liver volume was 1666 mL and the standard liver volume was 1590.9 mL. The volume of the right posterior lobe was 591 mL, accounting for 35.5% of the total liver volume, and the volume of the right hepatic liver accounted for 75% of the total liver volume (Fig. 5). An extended left hepatectomy was performed, and the portal vein was resected and reconstructed. The left hepatic vein was not infiltrated by the tumor while the venous wall of the middle hepatic vein was infiltrated by the tumor. Accordingly, the affected portion of the middle hepatic vein was resected, the left hepatic vein was dissected, and the middle hepatic vein was reconstructed by anastomosing the proximal left hepatic vein to the distal middle hepatic vein (Figs. 6, 7). The operation time was 620 min, the blood loss was 800 mL. The pathological findings showed: poorly differentiated intrahepatic cholangiocarcinoma, the resection margin of MHV was 1 cm from tumor, and the resection margin of PV was 0.8 cm from tumor. One metastasis was found in lymph node of No. 12. No metastasis was found in lymph node of No. 8, 9, 13a. The patient was followed up in outpatient clinic, he had recurrence in the omentum majus after 10 months of operation, and died after 18 months of operation with just supportive treatment.

### Patient 2

**Fig. 8:**
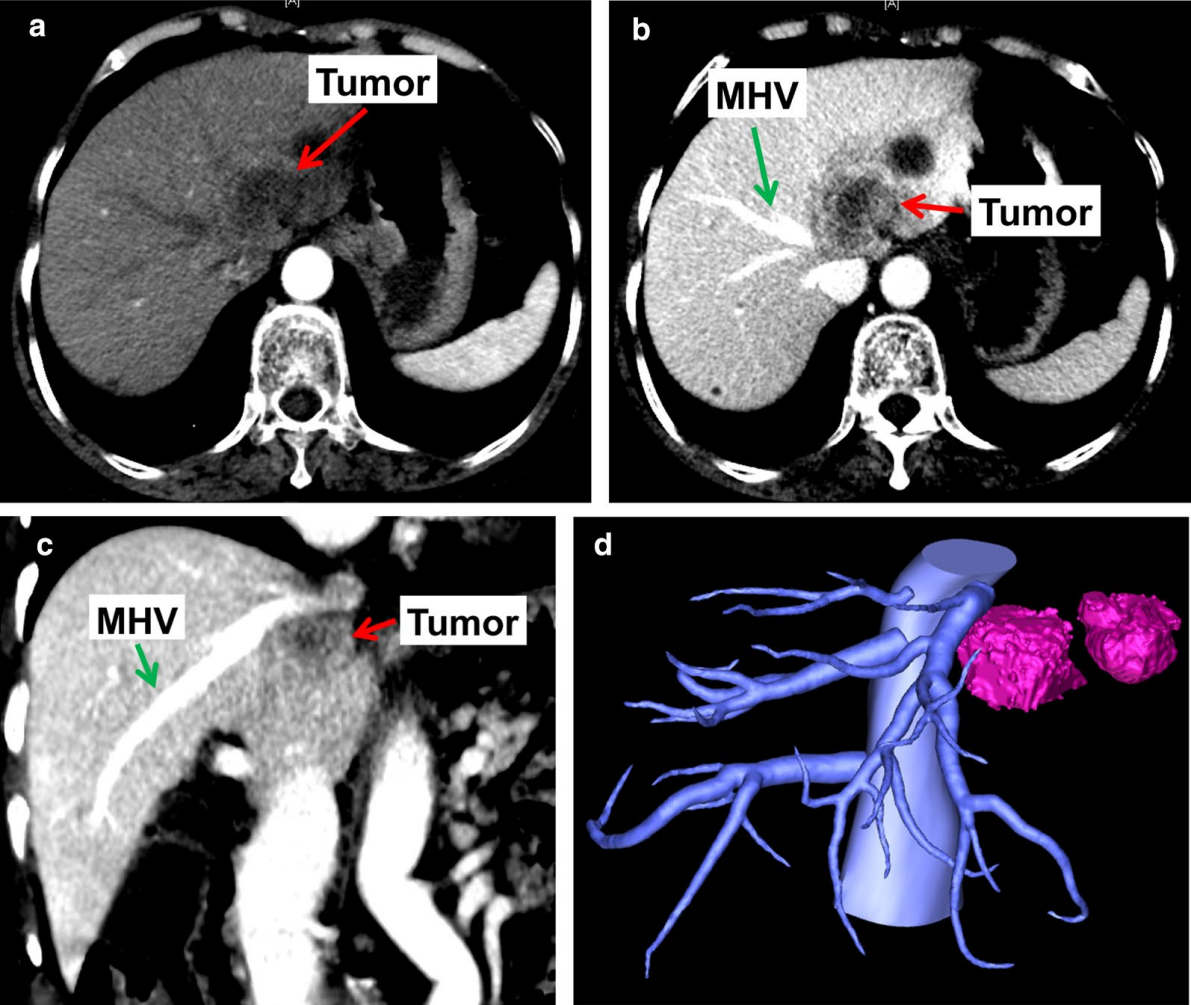
The intrahepatic cholangiocarcinoma was located in root of hepatic vein, which infiltrated the middle hepatic vein and the left hepatic vein. **a** Shows the location of tumor in artery phase. **b** Shows the relationship between tumor and MHV in transverse section. **c** Shows the relationships between tumor and MHV in coronal section. **d** The three-dimensional reconstruction of tumor and hepatic vein. *MHV* middle hepatic vein.

**Fig. 9:**
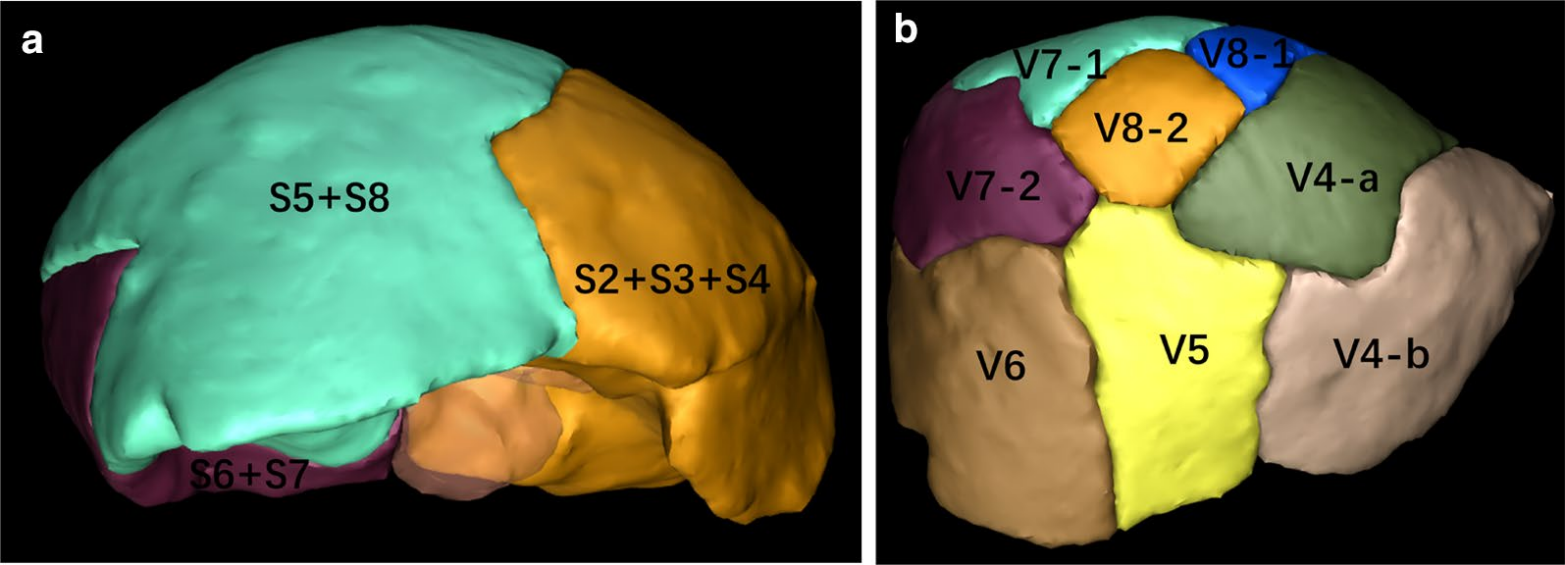
**a** Shows the area dominated by portal vein branch. The area dominated by portal vein branch: S2 + S3 + S4: 352 mL (36.0%), S5 + S8: 459 mL (47.0%), S6 + S7: 146 mL (14.9%). **b** Shows the area dominated by hepatic vein branch. The area dominated by hepatic vein branch: V4-a: 76 mL (7.9%), V4-b: 214 mL (22.4%), V5: 154 mL (16.1%), V8-1: 30 mL (3.1%), V8-2: 45 mL (4.7%), V6: 168 mL (17.6%), V7-1: 60 mL (6.3%), V7-2: 150 mL (15.7%).S2: segment 2, S3: segment 2, S4: segment 4, S5: segment 5, S6: segment 6, S7: segment 7, S8: segment 8, V4-a: hepatic vein of segment 4a, V4-b: hepatic vein of segment 4b,V5: hepatic vein of segment 5, V6: hepatic vein of segment 6, V7-1: a branch of RHV which drained part one of segment 7, V7-2: a branch of RHV which drained part two of segment 7, V8-1: a branch of MHV which drained part one of segment 8, V8-2: a branch of MHV which drained part two of segment 8. *MHV* middle hepatic vein, *RHV* right hepatic vein

**Fig. 10:**
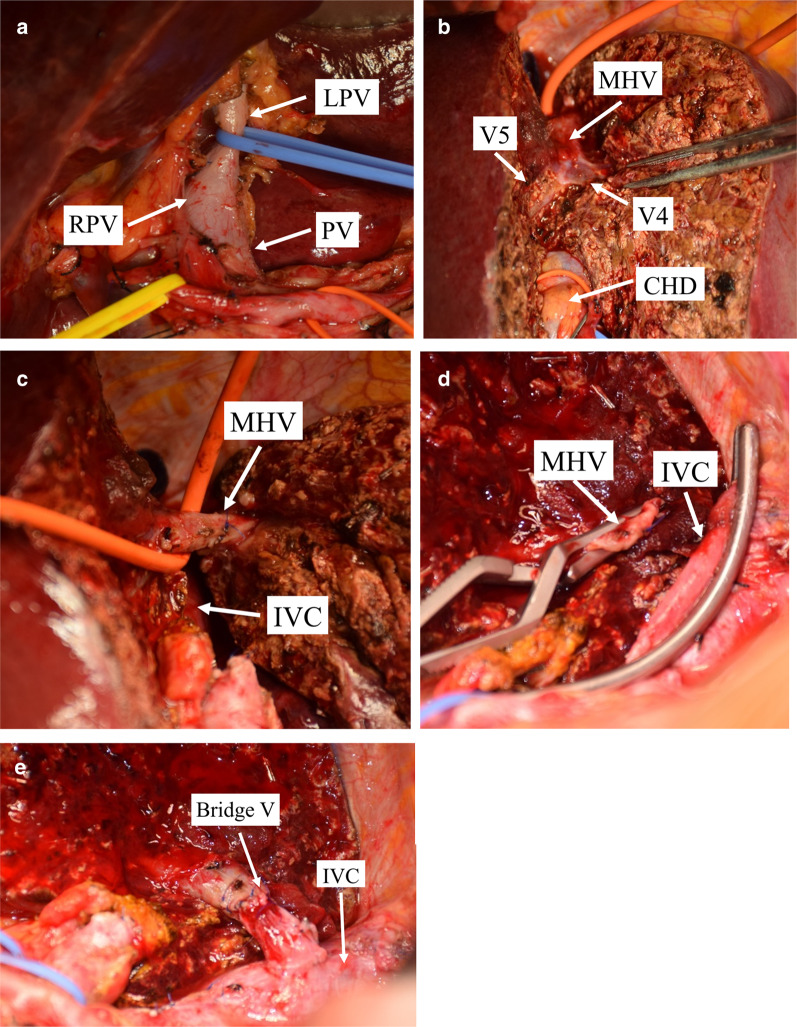
Intraoperative vascular operation: **a** dissected the first hepatic hilum, showing the left and right branches of the portal vein. **b** The trunk of middle hepatic vein and its branches. **c** Cut off the branch of the segment 4 originating from the middle hepatic vein. **d** The middle hepatic vein and the left hepatic vein were cut off from the root. The left liver lobe and the Spiegel lobe were removed to expose the inferior vena cava. **e** Using the left main branch of the portal vein as a vein graft to reconstruct the middle hepatic vein. *LPV* left portal vein, *RPV* right portal vein, *PV* portal vein, *MHV* middle hepatic vein, *CHD* common bile duct, *IVC* inferior vena cava, *Bridge V* bridge vein

**Fig. 11:**
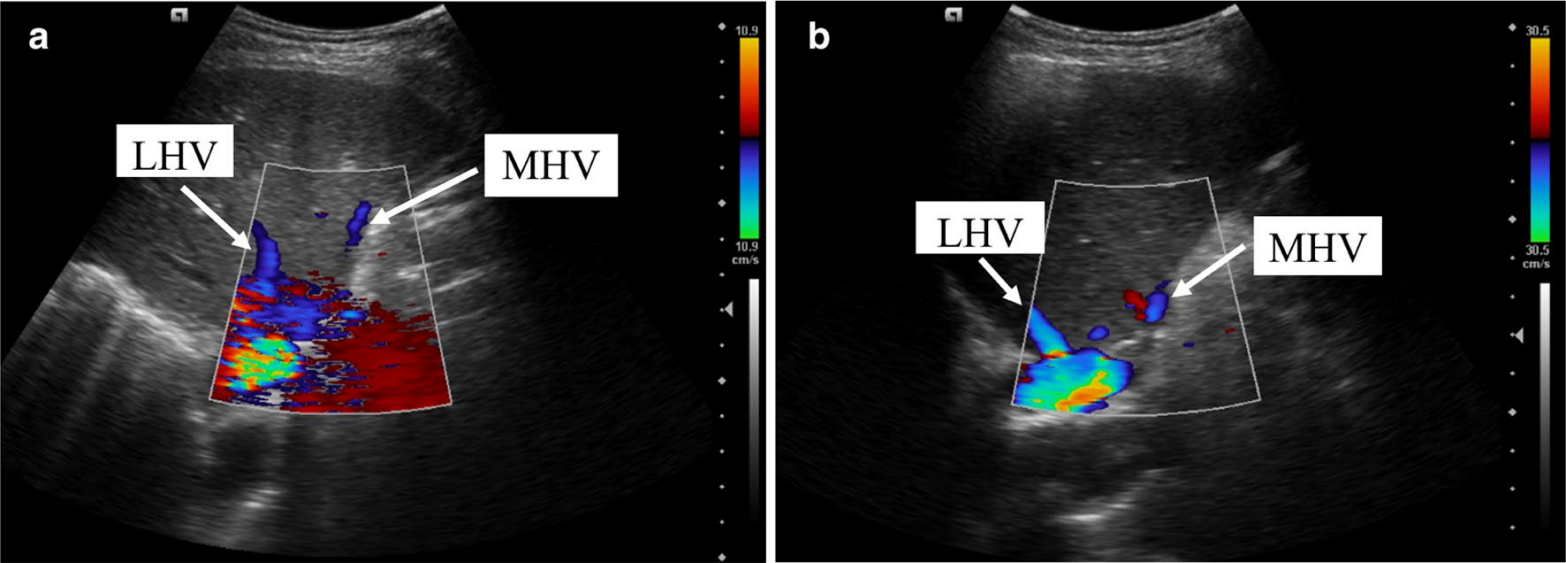
Ultrasound on postoperative day 4 (**a**) and postoperative day 60 (**b**). *MHV* middle hepatic vein, *LHV* left hepatic vein

The patient was a 69-year-old woman who had upper abdominal pain for 3 months prior. CT revealed “a lesion in the left lobe of the liver that was enhanced in the arterial and portal phases. The left and middle hepatic veins were infiltrated by a tumor (Fig. 8).” Blood tests indicated an alanine aminotransferase level of 13.4 U/L, an aspartate aminotransferase level of 18.5 U/L, an alkaline phosphatase level of 106.4 U/L, a gamma glutamyl transpeptidase level of 38.0U/L, a total bilirubin level of 14.08 umol/L, a direct bilirubin level of 4.70 umol/L, an albumin level of 47.8 g/L, a CA19-9 level of 104.09 U/mL, a CEA level of 6.07 ng/mL, and an AFP level of 4.82 ng/mL. The patient was diagnosed with intrahepatic cholangiocarcinoma. Preoperative liver function assessment indicated a Child–Pugh score of A, an ICG R15 of 2.4%, a standard liver volume of 1,039.1 mL, and a total liver volume of 957 mL. The volume of the right liver lobe was 605 mL, accounting for 58.2% of the standard liver volume and 63.2% of the total liver volume. The volume of the left liver lobe was 352 mL (Fig. 9). An extended left hepatectomy was performed. The location and orientation of the tumor were verified intraoperatively with ultrasound, and then the liver parenchyma was separated to isolate the middle hepatic vein from its root to its distal end. The left liver lobe and the affected portion of the middle hepatic vein were removed. The left portal vein was used as a vein graft to reconstruct the middle hepatic vein (Fig. 10). Postoperative ultrasound showed the reconstructed vein was patent (Fig. 11). The operation time was 600 min, the blood loss was 300 mL. The pathological findings showed: moderately differentiated intrahepatic cholangiocarcinoma, the resection margin of MHV was 1 cm from tumor, no microvascular invasion was seen. No metastasis was found in lymph node of No. 8, 9, 12, 13a. The patient was regularly followed in outpatient clinic and there was no recurrence evidence and complications. The follow-up was 2 years and 6 months until now.

## Discussion

Takahashi et al. found that liver function is closely related to blood flow [12]. It is important to ensure sufficient blood supply to the liver, but the patency of blood outflow from the liver is also very important. The major hepatic veins are often infiltrated by intrahepatic cholangiocarcinoma for the anatomical and pathological characteristics. If extensive liver resection for R0 resection results in liver blood outflow obstructed which leads to residual liver congestion, liver function is likely to diminish. So it is necessary to evaluate the area of hepatic vein preoperatively to decide whether and how to reconstruct the infiltrated hepatic vein.

The area of congested liver can be confirmed during surgery. Murata et al. found that when hepatic veins were occluded, the hepatic artery was the sole vessel for supplying blood to the liver, and the portal vein drained the congested portion of the liver [13]. Therefore, clamping a hepatic vein prior to resection and clamping the hepatic artery at the same time during surgery reveals the boundary of congested on the surface of the liver surface within 5 min [14]. The non-congested liver remnant volume can also be revealed during surgery with indocyanine green fluorescence imaging and hepatic vein clamping technique [15]. But this technique does not work sometimes. We have only one chance to use it during surgery [15]. When the hepatic veins are incompletely clamped, we cannot get the correct results. So, more liver remnant volume analysis methods are required, such as preoperative three-dimensional image reconstruction [14, 16]. The non-congested liver remnant volume can be determined by analyzing the volume of blood that each hepatic vein and its branches drains from each segment. With these methods, we can perform surgery safely by retaining enough liver volume which was normal on blood inflow and outflow.

Based on a study of living donor liver transplants, Sano et al. devised a criterion for hepatic vein reconstruction: the relevant volume of the congested liver should be subtracted from the remnant liver or the graft liver volume. When the remaining liver volume is less than 30% of the standard liver volume in normal liver resection or less than 40% in liver transplantation, reconstruction of the hepatic vein or its tributaries should be considered [14]. Based on a study of liver tumor resection, Mise et al. devised a criterion for hepatic vein reconstruction: venous reconstruction is recommended in patients with non-congested liver remnant volume smaller than 40% of total liver volume (TLV) when ICGR15 is less than 10% or non-congested liver remnant volume smaller than 50% of total liver volume when ICGR15 is 10–20% [16]. In some cases, liver volume increased because of continuous obstructive jaundice. So, for safety, we set 40% of both SLV and TLV as the cut-off value to reconstruct hepatic vein or its tributaries in liver tumor resection operation. When future non-congested liver remnant volume is smaller than 40% of SLV or TLV, hepatic vein reconstruction is needed to ensure blood outflow from the liver.

**Fig. 12:**
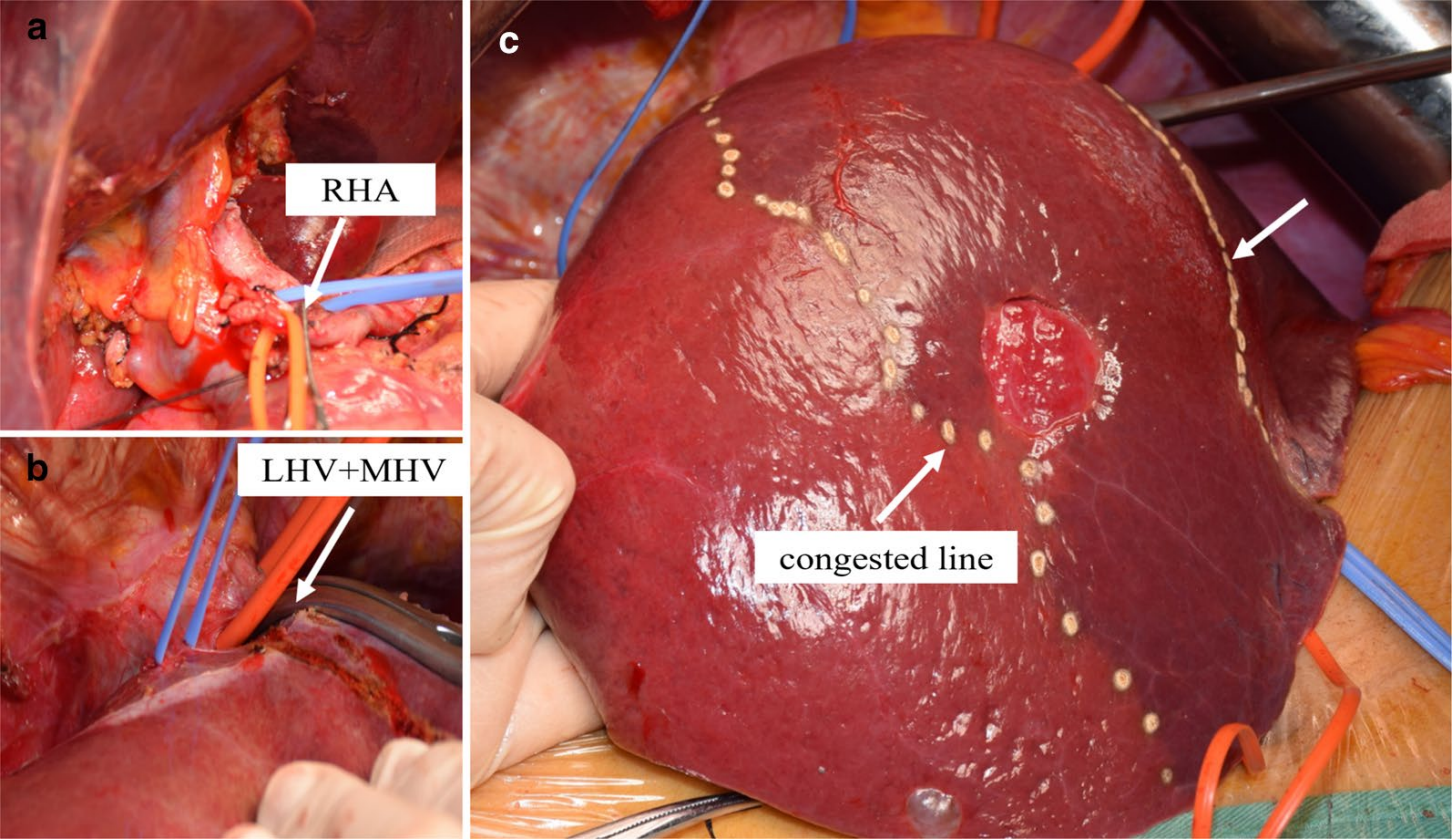
After blocking the right hepatic artery (**a**) and the middle and left hepatic veins (**b**), the hepatic congested area in segment 5 and segment 8 was revealed (**c**), indicating that this was the region drained by MHV. *MHV* middle hepatic vein, *LHV* left hepatic vein, *RHA* right hepatic artery

In Case 1, if the remnant liver is drained only by the right hepatic vein without reconstruction of the middle hepatic vein, the non-congested liver remnant volume is 630 mL, accounting for 37.8% of the total liver volume and 39.6% of the standard liver volume (Fig. 5). Diminished liver function can likely occur, so middle hepatic vein reconstruction must be factored into preoperative analysis. Blood outflow from the liver was ascertained postoperatively using ultrasound (Fig. 7), and liver function was normal. In Case 2, the volume drained by the right hepatic vein was 378 mL, accounting for 39.5% of the total liver volume and 36.4% of the standard liver volume (Fig. 9), so middle hepatic vein reconstruction was needed according to preoperative analysis. In case 2, we also confirmed the congested liver volume during surgery. The common trunk of the left and middle hepatic vein was clamped and then the right hepatic artery was clamped, revealing an area of congestion (Fig. 12) that was drained by the middle hepatic vein and that coincided with the area indicated by preoperative analysis. Hepatic vein reconstruction involving preoperative and intraoperative evaluation was required in both cases described in this report.

The vessel grafts used for hepatic veins reconstruction include autologous saphenous veins [1, 2], the internal jugular veins [3], the ovarian veins[4–6], the umbilical veins [6–8], artificial blood vessels [9], and allogeneic iliac vessels [10, 11]. When using a saphenous vein, the internal jugular vein, or the ovarian vein, a normal vessel needs to be harvested from the patient, thus increasing the number of surgical sites. An artificial blood vessel initially allows reconstruction of the hepatic vein, but it is less effective later on in comparison to a harvested vessel [9, 17]. Allogeneic blood vessels take time to acquire and cryopreserve. In the cases reported here, the vessels on the resected side of the liver were used as grafts to reconstruct the hepatic vein. The vessels used included the portal vein and a hepatic vein. The availability of blood vessels was assessed preoperatively, and the vascular grafts were harvested intraoperatively.

In Case 1, the tumor had not infiltrated the left hepatic vein and the tumor was sufficiently distant from the bifurcation of the left hepatic and middle hepatic veins, so the left hepatic vein was used to reconstruct the middle hepatic vein. Makuuchi et al. first reported a technique in which the left hepatic vein flap was used to repair the wall of the middle hepatic vein after the left hepatic vein and part of the lateral wall of the middle hepatic vein were removed because the left and middle hepatic veins had been infiltrated by a tumor [18]. The approach used in the current case differed slightly. Because the MHV was infiltrated by tumor, but the left hepatic vein was not infiltrated, so about 3 cm of the MHV trunk was resected from its root, and the trunk of the left hepatic vein was used to repair this part of MHV. In Case 2, the tumor had infiltrated the middle and left hepatic veins but was distant from the portal vein, so the left branch of the portal vein was used as a vessel graft to reconstruct the middle hepatic vein (Fig. 10). Ikegami et al. [19] and Junrungsee et al. [20] had reported MHV reconstruction with recipient's explanted portal vein in living donor liver transplantation. In this case, we reconstructed MHV with the trunk of portal vein in the resected liver itself.

In intrahepatic cholangiocarcinoma, the middle hepatic vein is usually infiltrated by a tumor at its central part. Therefore, the middle hepatic vein was first dissected from its root to the portion infiltrated by tumor and then dissected from its distal end to the tumor-infiltrated portion; this facilitates determining the length of middle hepatic vein trunk infiltrated by tumor and the specific location of the tumor, which is also good for bleeding control when dissociating liver parenchyma.

This report describes a method of harvesting a vascular graft from the portion of the liver that will be excised. Advantages of this approach include less damage than when using self-saphenous veins and self-internal jugular veins, highly similar characters than other grafts, obviates the need to wait for an allogeneic blood vessel. A limitation of this approach is that available blood vessels in the liver need to be assessed before removal.

With the development of surgical techniques, more extreme liver resection can be performed safely, especially for tumor which infiltrates key vessels. There are different vessel grafts with special advantages. We report two cases where vessel grafts from resected portion of liver were used, and find there are more advantages with good result. In future, more cases are needed to verify its effect.

## Data Availability

The authors declare that all the data in this article are available within the article.

## Abbreviations

CT: Computed tomography
TLV: Total liver volume
SLV: Standard liver volume
MHV: Middle hepatic vein
RABD: Bile duct of right anterior liver lobe
RPBD: Bile duct of right posterior liver lobe
RAA: Right anterior hepatic artery
RPA: Right posterior hepatic artery
RHA: Right hepatic artery
LPV: Left portal vein
LHV: Left hepatic vein
RPV: Right portal vein
PV: Portal vein
IVC: Inferior vena cava
Bridge V: Bridge vein
CHD: Common bile duct
ICG: Indocyanine green

## References

[1] Sozener U, Gulpinar K, Ozer Y (2014). Five right hepatic vein reconstructions using the autologous saphenous vein in the right lobe living-donor liver transplant: a case report. Exp Clin Transplant.

[2] Sakamoto Y, Yamamoto J, Saiura A (2004). Reconstruction of hepatic or portal veins by use of newly customized great saphenous vein grafts. Langenbecks Arch Surg.

[3] Shimagaki T, Yoshizumi T, Itoh S (2016). Liver resection with right hepatic vein reconstruction using the internal jugular vein: a case report. Surg Case Rep.

[4] Del Campo C (2000). Reconstruction of the hepatic and portal veins using a patch from the right ovarian vein. Am J Surg.

[5] Kubota K, Makuuchi M, Sugawara Y (1998). Reconstruction of the hepatic and portal veins using a patch graft from the right ovarian vein. Am J Surg.

[6] Miyazaki M, Ito H, Kimura F (2004). Hepatic vein reconstruction using autologous vein graft for resection of advanced hepatobiliary malignancy. Hepatogastroenterology.

[7] Takahashi M, Saiura A, Takahashi Y (2017). The usefulness of patch repair using the repermeabilized umbilical vein of the round ligament for hepatobiliary malignancies. World J Surg.

[8] Toshima T, Ikegami T, Matsumoto Y (2015). One-step venous reconstruction using the donor's round ligament in right-lobe living-donor liver transplantation. Surg Today.

[9] Orimo T, Kamiyama T, Yokoo H (2014). Usefulness of artificial vascular graft for venous reconstruction in liver surgery. World J Surg Oncol.

[10] Yamamoto M, Akamatsu N, Hayashi A (2017). Safety and efficacy of venous reconstruction in liver resection using cryopreserved homologous veins. J Hepatobiliary Pancreat Sci.

[11] Kaneoka Y, Yamaguchi A, Isogai M (2000). Hepatic vein reconstruction by external iliac vein graft using vascular clips. World J Surg.

[12] Takahashi H, Shigefuku R, Yoshida Y (2014). Correlation between hepatic blood flow and liver function in alcoholic liver cirrhosis. World J Gastroenterol.

[13] Murata S, Itai Y, Asato M (1995). Effect of temporary occlusion of the hepatic vein on dual blood in the liver: evaluation with spiral CT. Radiology.

[14] Sano K, Makuuchi M, Miki K (2002). Evaluation of hepatic venous congestion: proposed indication criteria for hepatic vein reconstruction. Ann Surg.

[15] Kawaguchi Y, Nomura Y, Nagai M (2017). Liver transection using indocyanine green fluorescence imaging and hepatic vein clamping. Br J Surg.

[16] Mise Y, Hasegawa K, Satou S (2011). Venous reconstruction based on virtual liver resection to avoid congestion in the liver remnant. Br J Surg.

[17] Yi NJ, Suh KS, Lee HW (2007). An artificial vascular graft is a useful interpositional material for drainage of the right anterior section in living donor liver transplantation. Liver Transpl.

[18] Hashimoto T, Kokudo N, Aoki T (2004). Reconstruction of middle hepatic vein using a rotating left hepatic vein flap. J Am Coll Surg.

[19] Ikegami T, Soejima Y, Taketomi A (2007). Explanted portal vein grafts for middle hepatic vein tributaries in living-donor liver transplantation. Transplantation.

[20] Junrungsee S, Lapisatepun W, Chotirosniramit A (2018). How to reconstruct middle hepatic vein branches with explanted portal vein and inferior mesenteric vein graft: a case report. Transplant Proc.

